# Late‐onset Wilson disease in older patient without ophthalmological findings, a case report

**DOI:** 10.1002/ccr3.2203

**Published:** 2019-05-16

**Authors:** Mehdi Yousefi, Masoud Yousefi, Anneh M. Gharravi

**Affiliations:** ^1^ Student Research Committee Shahroud University of Medical Sciences Shahroud Iran; ^2^ Department of Urology, Faculty of Medicine Mashhad University of Medical Sciences Mashhad Iran; ^3^ School of Medicine Shahroud University of Medical Sciences Shahroud Iran

**Keywords:** copper, liver, older, patient, Wilson disease

## Abstract

A 60‐year‐old man was referred to hospital for loss of consciousness. Her late‐onset Wilson disease was diagnosed with elevated serum bilirubin, hepatic copper concentration, and urinary copper excretion. Zinc sulfate administration decreased the majority of clinical symptoms.

## 1 INTRODUCTION

Wilson disease (WD) is an autosomal recessive inherited disorder of liver copper metabolism with accumulation of this metal in many organs predominantly in the liver and brain.^1^

The gene ATP7B on chromosome 13 is responsible for copper's transfer. WD is caused by mutations of gene ATP7B, and the age of onset varies from 3 to 50 years.

The copper deposition in WD can cause neurologic, mental diseases or destruction of the liver. Liver diseases, including recurrent jaundice, hepatitis, and hepatobiliary failure, are usually the first symptoms of WD in children and young people. Older age patients usually have no liver problems.

As the laboratory findings are specific for WD, the diagnosis is made with the ceruloplasmin level, the amount of urine, or blood copper. Ophthalmic examination and liver function and genetic testing help to establish the diagnosis.^2^

Many cases of WD occur in adolescents, and in the geriatric age it is rare.^3^ The disease affects men and women equally,^4^ and many cases of WD present symptoms at <40 years of age.

In the present case report, we describe an unusual case of late‐onset WD in a 60‐year‐old patient.

## 2 CASE PRESENTATION

### 2.1 Ethics

Written informed consent was obtained from the patient. The patient verbally consented to the use of his clinical images for this report.

### 2.2 History

A 60‐year‐old man was referred to Imam Reza hospital of Mashhad for loss of consciousness. The patient of the present case had no history of severe cirrhotic change before admission to hospital.

A review of the patient history did not reveal previous or current history of similar illness in his siblings and close contacts. Both his parents were Iranian, of the Fars ethnic group from northeast Iran.

### 2.3 Clinical finding and physical examination

On examination, he was afebrile with a normal blood pressure measuring 125/80 mm Hg and heart rate of 80 beats/min. Physical examination supports the diagnosis of hepatic encephalopathy.

1. Hepatic presentation

After admission, he developed hepatic encephalopathy and cirrhosis with abnormal liver function.

2. Neurologic presentation

After admission, patient was conscious and well oriented.

3. Ophthalmic presentation

The patient had normal ocular results without Kayser‐Fleischer rings (KF rings).

4. Psychiatric presentation

His neurologic status was unremarkable.

5. Other organs

Kidney function tests were normal. Central nervous system examination showed normal higher mental functions.

### 2.4 Laboratory findings

Laboratory studies revealed abnormal liver function, including an elevated serum total bilirubin (T‐Bil) level of 2.06 mg/dL (upper limit of normal [ULN]: 1.2 mg/dL), direct bilirubin level of 0.55 mg/dL (ULN: 0.25 mg/dL), an elevated liver enzymes SGOT level of 45 IU/L (ULN: 31 IU/L), ALK phosphatase level of 588 (ULN: 306 U/L) with hypoalbuminemia (Serum Albumin‐3.3 g/dL; lower limit of normal [LLN]: 3.5 g/dL).

Elevated urinary copper excretion (270 μg/24 h) observed (ULN: 70 μg/24 h) in urine biochemistry.

Ceruloplasmin level in patient was 221.9 mg/L (LLN: 150 mg/L, ULN: 300 mg/L). Serum ceruloplasmin concentration was measured by using a nephelometric method. In the coagulation profile, prothrombin time (PT) level was 15.5 seconds (LLN: 10.5, ULN: 13 seconds) with partial thromboplastin time (PTT) level of 43 seconds (LLN: 28, ULN: 45 seconds), elevated international normalized ratio (INR) level of 1.4 ratio (ULN: 1 ratio) and fibrinogen level of 1941 mg/dL (LLN: 150, ULN: 350 mg/dL).

Also, low level of ammonia (128 µg/dL) was detected (LLN: 130, ULN: 145 µg/dL). The patient tested negative for hepatitis B virus (HBV) by TaqMan real‐time PCR. Antinuclear antibody (ANA) was 5 U/mL (LLN: 10 U/mL) when measured with immunochemiluminescenc procedure, and HCV Ab test was negative.

### 2.5 Imaging diagnosis

#### 2.5.1 Ultrasonography

**Figure 1:**
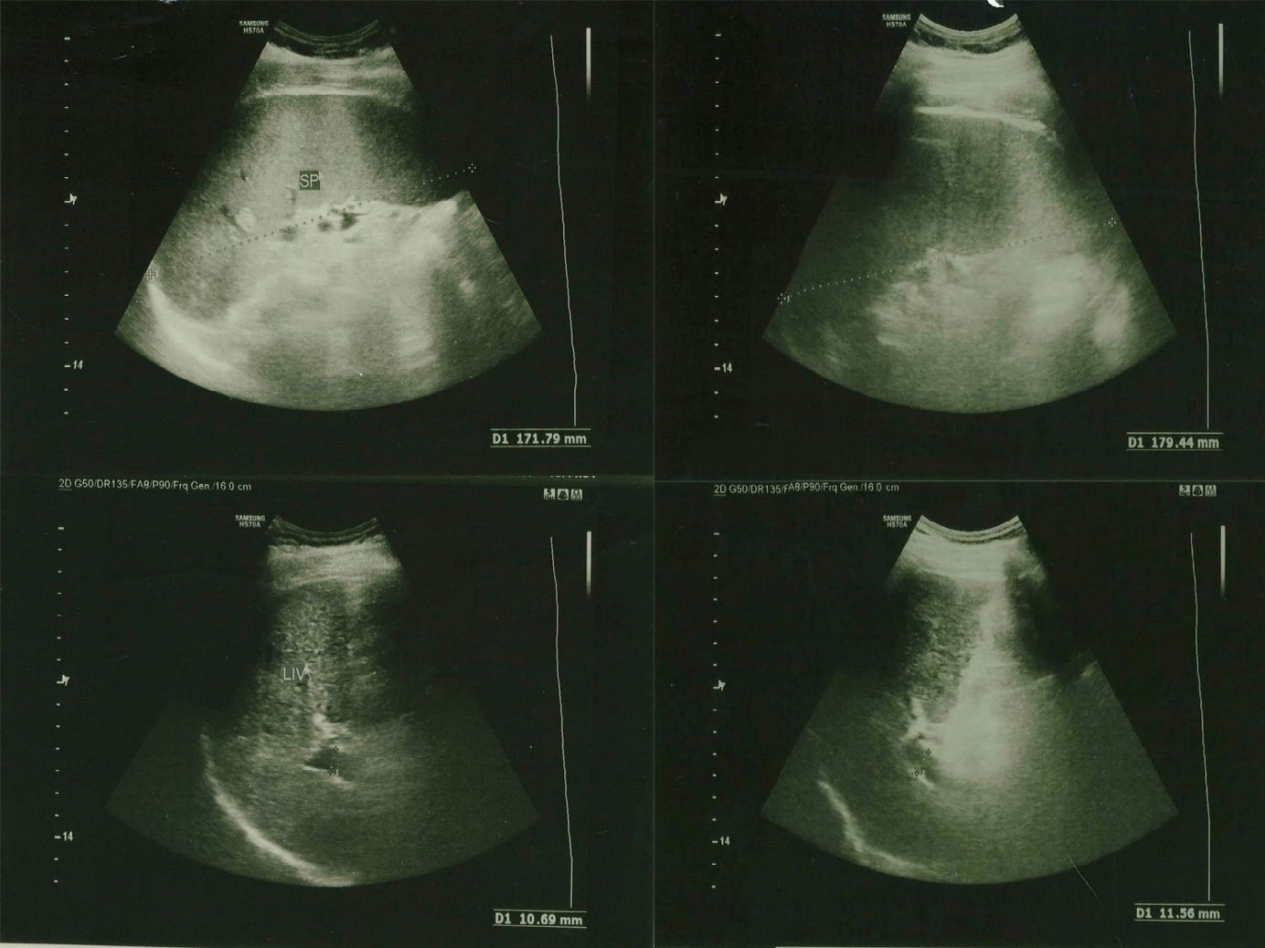
Ultrasonography of liver and spleen. Splenomegaly was detected

**Figure 2:**
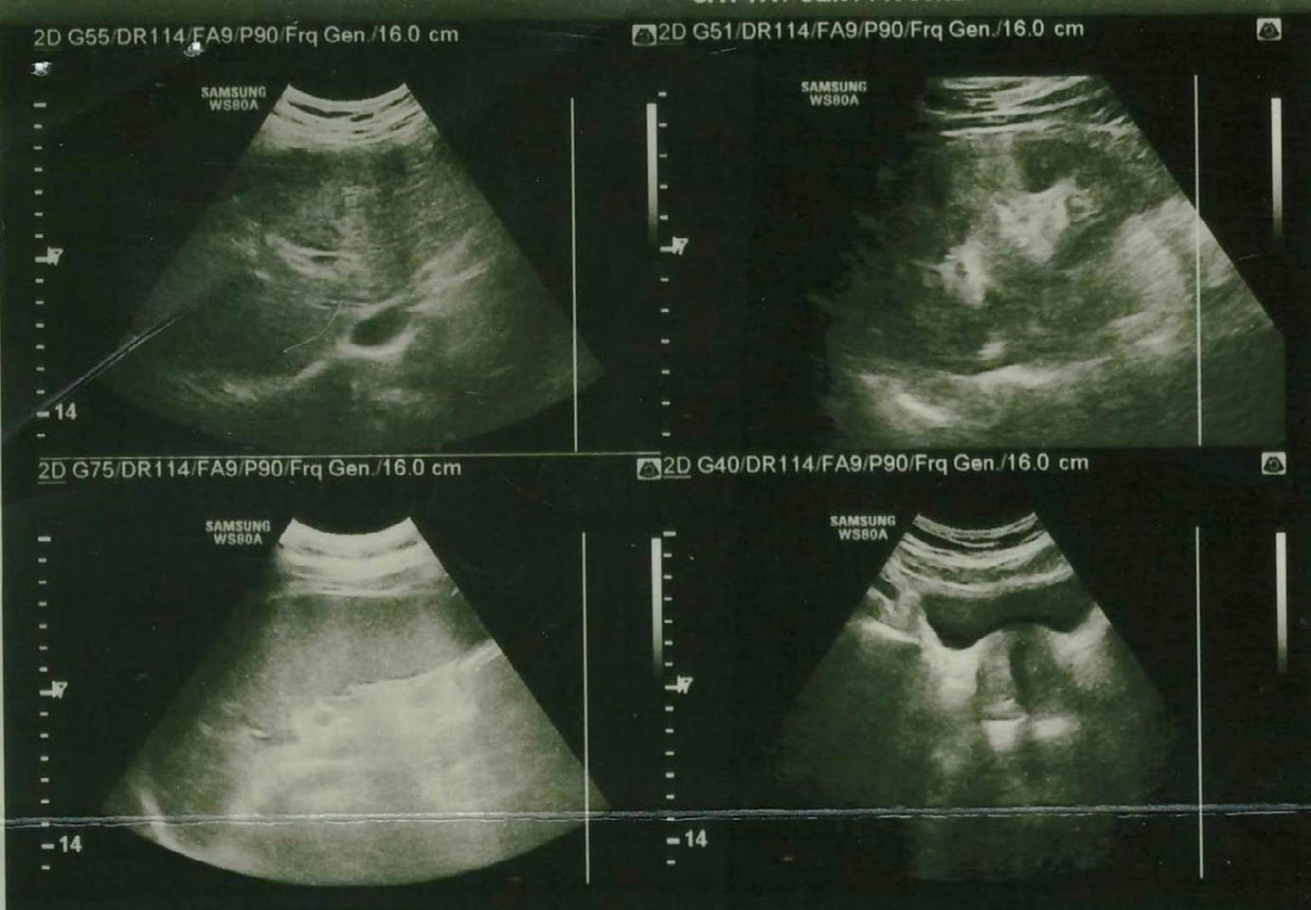
Ultrasonography of liver and normal portal vein diameter

Ultrasonography of the abdomen revealed features suggestive of chronic liver disease and splenomegaly (longitudinal diameter 179 mm). The gallbladder was not visualized due to previous cholecystectomy. Normal size of portal vein diameter and common bile duct (CBD detected, respectively, 11 and 4 mm (Figures 1 and 2). The present case had no history of severe cirrhotic change before admission to hospital.

#### 2.5.2 Endoscopic examination

**Figure 3:**
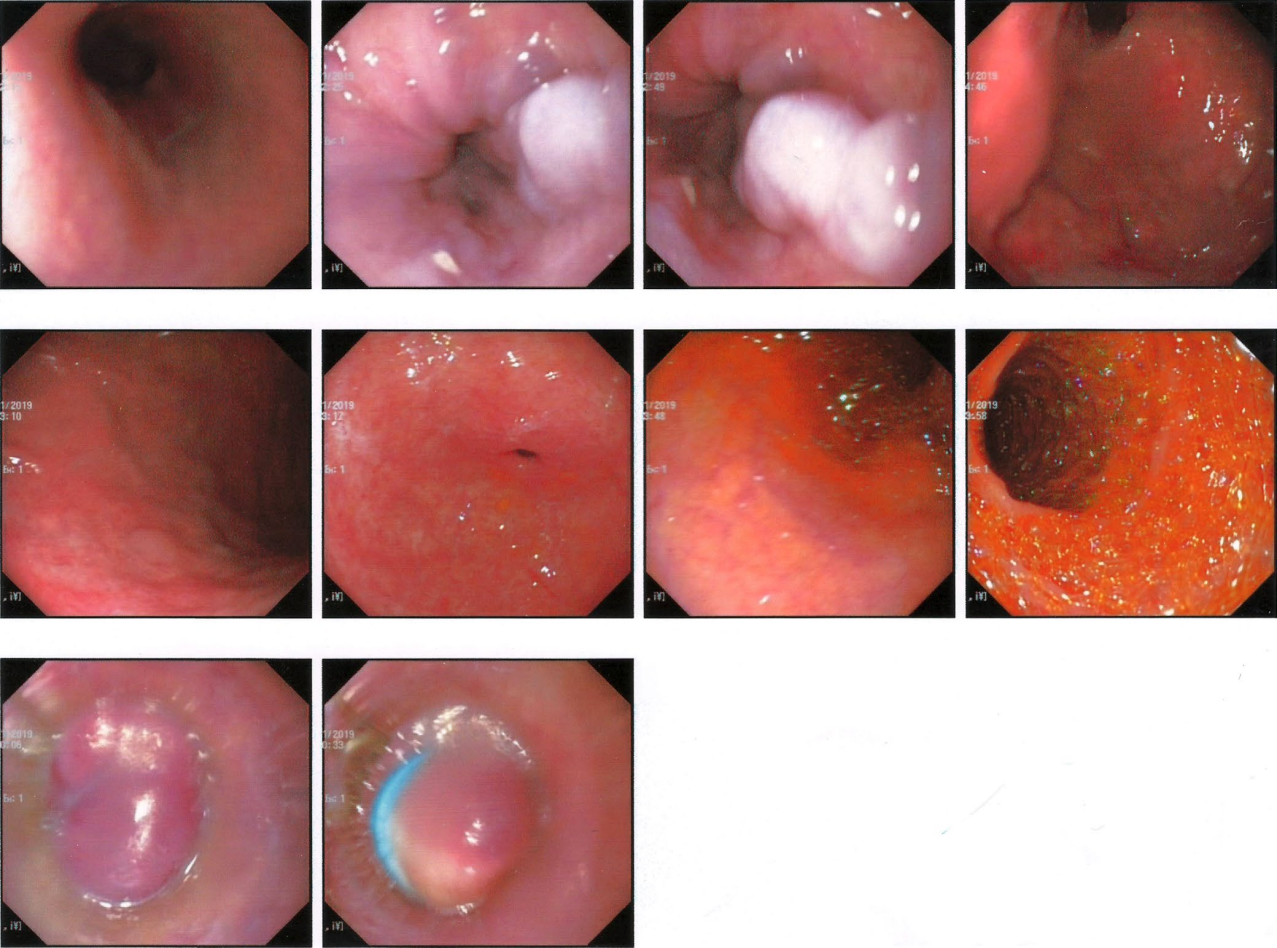
Endoscopic of middle and lower esophagus showing varices at distal esophagus

Endoscopic examination showed 2‐3 rows varices at the distal esophagus and proximal lesser curvature (Figure 3).

### 2.6 Pathological diagnosis

#### 2.6.1 Liver biopsy and histological examination

Liver biopsy revealed cirrhotic change with moderate to severe steatosis, portal inflammation, liver cell degeneration and necrosis, and glycogenation of periportal hepatocytic nuclei.

Masson's trichrome stain revealed the collagenous fibers surrounding nodules of hepatocytes.

For liver copper concentration detection, liver sample was obtained by needle biopsy. The liver copper concentration in the present case was 1016 mcg/g dry weight liver when measured by neutron activation analysis.

#### 2.6.2 Transient elastography (fibroscan) assessment

Background patient liver parenchyma showed heterogeneously fibrotic from 19 to 45 kilopascals (kPa). The median fibrosis was 35.5 kPa (equal to F4 based on a Metavir histological index).

**Figure 4:**
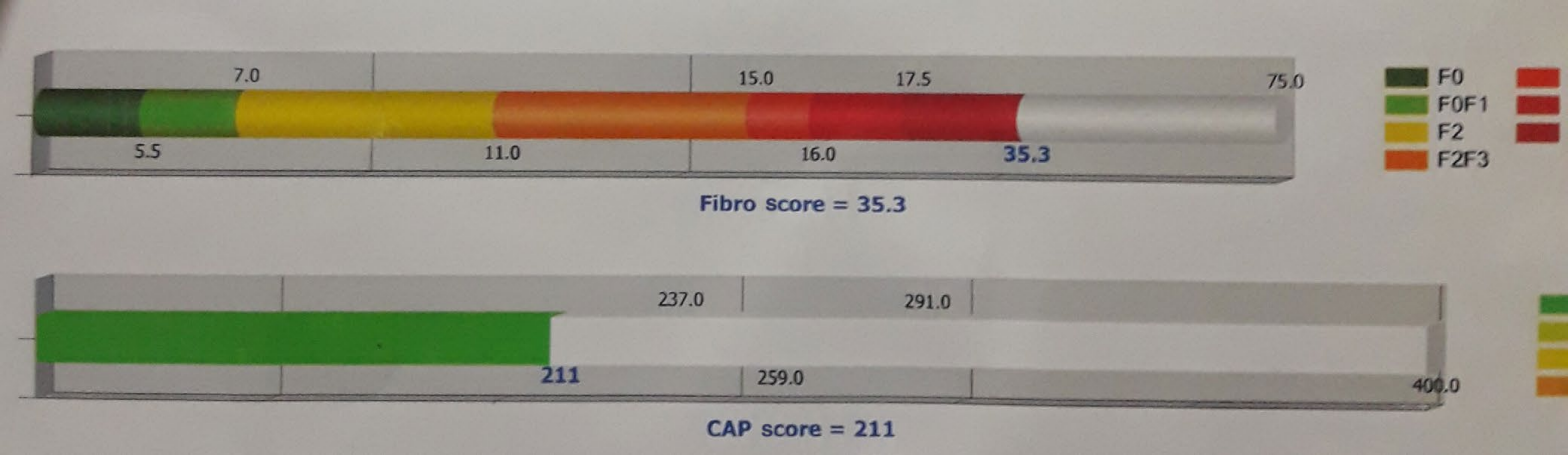
FibroScan assessment of liver showing heterogeneously fibrotic liver

The controlled attenuation parameter (CAP) score for liver steatosis was 211 db/m (Figure 4).

### 2.7 Differential diagnosis

The liver of the patient showed chronic liver disease symptoms in which another cause has not been established. Therefore, the diagnosis of WD was considered.

### 2.8 Treatment

After diagnosis of WD, the patient was prescribed Pantazol (40 mg/d), Amilodipin (5 mg/d), and zinc sulfate (150 mg/d) on day 9 of hospitalization. During the treatment, the diet of the patient was restricted to low‐copper, high‐calorie, and low‐protein meals.

### 2.9 Follow‐up and outcome

After treatment, central nervous system examination showed normal higher mental functions. Other systemic examination was normal. Hepatic copper measurement showed decreased hepatic copper concentration. The use of zinc significantly improved the majority of clinical symptoms of WD. The patients completely responded to the therapy, at the end of the follow‐up.

## 3 DISCUSSION

WD is a rare autosomal recessive disorder of copper transport. The excessive deposition of copper due to decreased transport into bile thereby leads to liver damage. The onset of Wilson disease at <40 years of age is rare. We report a unique case of late‐onset WD in older patient with typical biochemical markers and absence of typical ocular findings.

In the present case, a fourfold increase of liver copper concentration is considered as the best available test for diagnosis of WD. The accuracy of the test was assessed by other assessments. In literature, a copper content of liver more than 250 mcg/g dry weight is considered diagnostic for WD.^5^ The liver has potential to store excess copper in younger individuals than adults.^6^

In the recent years, the use of FibroScan to assess liver fibrosis in WD Patients is becoming more widespread. In the literature, a value higher than 8.4 kPa is indicative of severe fibrosis. In the present case, a fourfold increase of the value was considered as the best available test for diagnosis of chronic liver disease.^7^ Therefore, FibroScan values can be clinically useful for predicting fibrosis stages of WD patients.^8^

In the present case, presence of esophageal varices correlated significantly with the other signs of WD. Esophageal varices are one of a serious consequences of portal hypertension, which usually have no signs and symptoms until bleeding. Loss of consciousness is one of the most signs and symptoms of esophageal varices bleeding.^7^, ^9^, ^10^

In the present case report, ceruloplasmin level was 221.9 mg/L (in the normal range). Previous studies indicated that, in patients with hepatic WD, plasma ceruloplasmin is often in the normal range.^2^

While typically the diagnosis of WD is based on the presence of ophthalmological findings, low level of ceruloplasmin and elevated urinary copper excretion, it is a rare event for WD to have onset in geriatric age. However, in the present case, with elevated urinary copper excretion and hepatic copper deposition, ceruloplasmin and ophthalmological finding were normal. Also, previous cases showed that common neurologic symptoms of WD appear and progress with time.

But in the present case central nervous system examination was completely normal.

The approach to treatment of WD depends on clinical presentation at diagnosis and remains controversial. The current approach includes (a) chelators (D‐penicillamine and trientine) for the excretion of copper from the body and (b) zinc salts to reduce copper absorption.^11^ In the present case, Zinc sulfate administration (150 mg/d) decreased the majority of clinical symptoms. Previous studies revealed that Zinc sulfate administration is effective in WD patients with mild liver disease.^12^

Studies conducted on WD patients indicated that the Zinc sulfate administration is safe and inexpensive.^13^ In the present case, the normalization of liver enzymes was observed after zinc therapy.^12^

## 4 CONCLUSIONS

Typically, the diagnosis of WD is based on the presence of ophthalmological findings, low level of ceruloplasmin and elevated urinary copper excretion. But in some case of WD ophthalmological finding can be normal.

The result from this case is in consistent with previous studies that in patients with hepatic findings, WD should remain a diagnostic possibility.

## CONFLICT OF INTEREST

The author(s) declared no potential conflicts of interest with respect to the research, authorship, and/or publication of this article.

## AUTHOR CONTRIBUTIONS

M Yousefi, M Yousefi and AM Gharravi: involved in the literature review and creation of the manuscript. M Yousefi, M Yousefi, and AM Gharravi: involved with editing the manuscript.

## STATEMENT OF ETHICS

Written informed consent was obtained from the patient. The patient verbally consented to the use of his clinical images for this report.
